# Research on the Effects of Drying Temperature on Nitrogen Detection of Different Soil Types by Near Infrared Sensors

**DOI:** 10.3390/s18020391

**Published:** 2018-01-29

**Authors:** Pengcheng Nie, Tao Dong, Yong He, Shupei Xiao

**Affiliations:** 1 College of Biosystems Engineering and Food Science, Zhejiang University, Hangzhou 310058, China; npc2012@zju.edu.cn (P.N.); 21613052@zju.edu.cn (T.D.); xsp941230@163.com or 180312@zju.edu.cn (S.X).; 2 Key Laboratory of Sensors Sensing, Ministry of Agriculture, Zhejiang University, Hangzhou 310058, China; 3 State Key Laboratory of Modern Optical Instrumentation, Zhejiang University, Hangzhou 310058, China

**Keywords:** nitrogen, near infrared sensors, drying temperature, SPA-MLR, PLS, CARS

## Abstract

Soil is a complicated system whose components and mechanisms are complex and difficult to be fully excavated and comprehended. Nitrogen is the key parameter supporting plant growth and development, and is the material basis of plant growth as well. An accurate grasp of soil nitrogen information is the premise of scientific fertilization in precision agriculture, where near infrared sensors are widely used for rapid detection of nutrients in soil. However, soil texture, soil moisture content and drying temperature all affect soil nitrogen detection using near infrared sensors. In order to investigate the effects of drying temperature on the nitrogen detection in black soil, loess and calcium soil, three kinds of soils were detected by near infrared sensors after 25 °C placement (ambient temperature), 50 °C drying (medium temperature), 80 °C drying (medium-high temperature) and 95 °C drying (high temperature). The successive projections algorithm based on multiple linear regression (SPA-MLR), partial least squares (PLS) and competitive adaptive reweighted squares (CARS) were used to model and analyze the spectral information of different soil types. The predictive abilities were assessed using the prediction correlation coefficients (R_P_), the root mean squared error of prediction (RMSEP), and the residual predictive deviation (RPD). The results showed that the loess (R_P_ = 0.9721, RMSEP = 0.067 g/kg, RPD = 4.34) and calcium soil (R_P_ = 0.9588, RMSEP = 0.094 g/kg, RPD = 3.89) obtained the best prediction accuracy after 95 °C drying. The detection results of black soil (R_P_ = 0.9486, RMSEP = 0.22 g/kg, RPD = 2.82) after 80 °C drying were the optimum. In conclusion, drying temperature does have an obvious influence on the detection of soil nitrogen by near infrared sensors, and the suitable drying temperature for different soil types was of great significance in enhancing the detection accuracy.

## 1. Introduction

Soil, which provides nutrients in the process of plant growth, is the foundation and plays an important role in agriculture. Thus, it is of great importance to obtain soil nutrient elements such as soil nitrogen quickly and accurately for precision fertilization and agricultural production [1,2]. Many conventional soil analytical techniques such as Dumas combustion are often complex, with multi-component interactions [3]. At present, near infrared sensors (NIR) have been successfully applied to the fields of agriculture, food, medicine, petroleum and chemistry, and are one of the most important analytical methods, indispensable in qualitative and quantitative analysis [4,5]. In recent years, many scholars have used near infrared sensors to detect soil nitrogen and improved the detection accuracy in the aspects of soil pretreatments, spectral data processing, characteristic band selection and algorithm optimization. 

Dalal predicted soil nitrogen with the multiple linear regression method at the spectral bands of 1702 nm, 1870 nm and 2052 nm [6]. Lee et al. found that the sensitive bands of soil total nitrogen were not only affected by soil type, but also determined by sampling depth [7]. He et al. detected N, P, K, organic matter (OM) and pH content in a loamy mixed soil by NIR. The results showed that the correlation coefficient between measured and predicted values of N, OM and pH were 0.93, 0.93 and 0.91, respectively, but were not suitable for predicting P and K [8,9]. Stoner et al. described the spectral reflectance curves and their relationships with five soil types in detail according to the characteristics of the spectral reflectance of surface soil samples from the United States and Brazil [10]. Ramirez et al. analyzed the soil organic matter, total nitrogen, organic carbon, clay and calcium. The results suggested that the forecast equation spectrum of soil organic matter and total nitrogen performed better [11]. Brunet’s research pointed out that the grinding treatment of soil reduced the prediction accuracy of organic carbon, while grinding had no obvious effects on total nitrogen detection [12]. Barthès et al. studied how the NIR prediction of carbon and nitrogen content were affected by sample grinding, drying and replication. The results showed that the prediction accuracy was the highest with oven-dried, 0.2 mm ground soil samples [13]. Hernandez et al. found that the soil particle size and high water content would affect the accuracy of soil organic nitrogen prediction [14]. Nie’s research indicated that the soil with the strictest pretreatment (dried, ground, sieved and pressed) had the highest accuracy in predicting the soil nitrogen content [15]. Cozzolino et al. found that the prediction correlation coefficients among coarse sand (2–0.25 mm), fine sand (0.25–0.05 mm) and clay sand (<0.05 mm) were 0.90, 0.92 and 0.96 respectively based on NIR [16]. Nocita et al. used visible near infrared sensors to detect soil organic carbon content under different soil moisture gradients. The soil samples were collected and dried, and then 0.005 grams of water was added to each soil sample. It was found that water content could affect the detection accuracy, and the R^2^ of the optimum model was 0.74 [17,18,19].

However, there is little research about the influence of soil drying temperature on soil nitrogen detection by near infrared sensors. Soil texture, soil moisture content and external temperature all affect soil nitrogen detection [20,21]. Particularly, the temperature has an influence on the water removal and activity of urease [22]. Coarse samples were dried at 20 °C for 48 h for the prediction of C and N content using visible near infrared spectroscopy and the comparative method was used to detect their potential mineralization in heterogeneous soil samples [23]. The flat-dry samples were dried at 35 °C for 12 h to explore the effects of soil sample pretreatments and standardized rewetting [24]. Soil was subjected to oven drying at 60 °C for 24 h, after which they were ground and sieved with a 0.002 m sieve [25] or soil samples were dried at 95 °C for 24 h [26] and then sieved for estimating total nitrogen using NIR. Besides this, soil samples were dried at 80 °C for 8 h to detect soil nitrogen with different pretreatments using NIR on He’s research [27]. Moreover, soil samples were dried naturally, rolled and broken into pieces, then sieved with a 2-mm screen to estimate the organic matter or soil properties using NIR [28,29]. However, the impact of drying temperature on soil nitrogen detection has been studied little and the mechanism was not clear yet. 

The objective of this study was to investigate the influence of temperature on soil nitrogen detection of loess, calcium soil and black soil as well as find the suitable drying temperature for different soil types to enhance the nitrogen detection accuracy by near infrared sensors. Besides this, this study compared the differences of the near infrared spectra of different soils and different temperatures, and the prediction models established by SPA-MLR, PLS and CARS were analyzed from the perspective of temperatures, soil properties and algorithms.

## 2. Materials and Methods

### 2.1. Experimental Materials and Sample Preparation

The experimental soils included black soil, loess and calcium soil, which were collected from different regions with different physical and chemical properties in China. Among them, black soil comes from the Greater Khingan region, whose pH value is neutral to slightly alkaline. Loess comes from Xi’an, Shanxi province, whose soil properties are loose and porous. Calcium soil with the features of loose and poor structure is from Jinan, Shandong province. The soil sample preparation process was as follows. First, the soil samples were sieved with a 40 mesh sieve (0.425 mm) and grinded; in addition, the urea solutions with different concentrations were prepared. Second, different nitrogen concentration gradients for three kinds of soils were prepared, that were, loess (0.09–0.93 g/kg, 0.1 g/kg per gradient), calcium soil (0.32–1.17 g/kg, 0.1 g/kg per gradient), black soil (0.46–2.15 g/kg, 0.2 g/kg per gradient). Meanwhile, the three kinds of soils without urea added were set as references. There were 16 samples for each concentration, and each soil type contained 11 nitrogen gradients. Third, the experiments were carried out in four groups, each group containing three soil types. Black soil, loess and calcium soil were dried after 50 °C for 24 h (group I), 80 °C for 18 h (group II) and 95 °C for 12 h (group III) respectively. Other soil samples were dried and then placed at 25 °C for 12 days (group IV).

### 2.2. Spectrometric Determination

**Figure 1:**
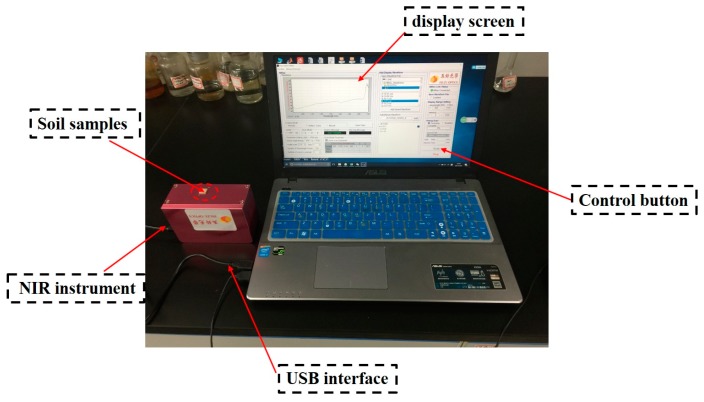
Near infrared (NIR) spectrum soil detection platform.

The portable near infrared optical instrument is from Isuzu Optics Corp (Shanghai, China). It is an interferometer instrument which is reflective with two integrated tungsten halogen lamps. The instrument collects spectral information in the range of 900–1700 nm, whose optical resolution is 10 nm and the signal-noise ratio is 5000:1 in a 1 s scan; the size is 120 × 85 × 54 mm and the weight is 900 g. The soil detection platform is shown in Figure 1.

When the spectrum of soil were measured, the samples were placed on the light source window, which avoided the phenomenon of light leakage since the size of the soil sample is larger than that of the light source window. Before performing the spectroscopic measurement, the instrument should be preheated for 15 min and be prepared with blackboard and whiteboard correction operation. In order to maintain the integrity of the original soil spectra and the rapidity of the detection process, the spectral acquisition parameter is set up as 400 points, and the spectrum is obtained by averaging three scans.

### 2.3. Data Analysis

Near infrared light is an electromagnetic wave between the infrared and visible light whose wavelength range is from 780 nm to 2526 nm [30]. The spectral information originates from the vibration of the O–H, C–H and N–H groups, which can reflect the variety of organic matter in the characteristic signal of the spectral region [31]. According to Lambert absorption law [32], the spectral characteristics would change as material composition or structure changes. However, at the same time, it can also be affected by the soil surface texture, density and uneven distribution of internal components, which is very difficult for all the redundant information of the spectral data to be eliminated, such as the overlap. Therefore, in order to achieve the purpose of qualitative or quantitative analysis of complex mixtures, it is necessary to extract and analyze the weak chemical information by chemometrics method in the spectral analysis. 

In this paper, the original spectra were preprocessed by Savitzky–Golay (S-G) smoothing. Then three modeling methods were used to model and analyze the spectral information. The SPXY method [33] was used to divide the three soil samples into two groups according to the proportion of 2:1, among which 118 soil samples (N1) were calibrated and 58 soil samples (N2) were validated at different temperatures and different soils. All data analysis was based on MATAB R2014a (The Math-Works, Natick, MA, USA).

### 2.4. Spectral Preprocessing Method

Savitzky–Golay (S-G) smoothing [34], also known as polynomial smoothing, uses the weighted average method to quantize the data in the moving window by polynomial least squares fitting as well as emphasizing the central role of the center point. The formula of average wavelengths after S-G smoothing is

(1) $$x_{k,smooth}=\bar{x_{i}}=\frac{1}{H}\overset{+w}{\underset{i=-w}{\sum }}x_{k+1}hi$$

where H is the normalization factor, hi is the smoothing coefficient and $H=\sum_{I=-W}^{+W}h_{i}$. The measured value multiplied by the smoothing coefficient minimizes the smoothing influence on the useful information. In the experiment, the S-G was used to remove the background noise of the instrument and the noise of the spectrum.

### 2.5. SPXY Method

SPXY, the method of choosing the calibration sample, was put forward on the basis of KS methods by Galvao et al. [35]. The basic principle is that spectrum and concentration variables are considered at the same time to calculate the distance of the samples, the distance formula is as follows:

(2) $$d_{xy}=\frac{d_{x}\left(i,j\right)}{max_{i,j\in \left(1,z\right)}\left[d_{x}\left(i,j\right)\right]}+\frac{d_{y}\left(i,j\right)}{max_{i,j\in \left(1,z\right)}\left[d_{y}\left(i,j\right)\right]},i,j\in \left[1,z\right]$$

In the formula, $d_{x}\left(i,j\right)$ is based on spectral characteristic parameters for the calculation of the distance between the samples, while $d_{y}\left(i,j\right)$ is based on concentration characteristic parameters for the calculation of the distance between the samples—which makes the sample in spectrum space and concentration space have the same weightiness—divided by their corresponding maximum standardizing, respectively. *z* is spectral space.

### 2.6. Modeling Method

#### 2.6.1. Partial Least Squares Method

Partial least squares regression (PLSR) is one of the most widely used methods for quantitative correction in chemometrics. In the PLS model, the principal components of the matrix *X* and the matrix *Y* are decomposed in order to extract the most comprehensive variables with respect to the dependent variables and maximize the correlation between the principal component and the concentration, which overcomes the negative effects of the multiple correlation of variables and further improves the reliability of the model [36]. In this paper, the whole band spectral data are used as independent variable *X*, and the nitrogen content are considered as the dependent variable *Y*. The minimum cross validation is used to verify the root mean square error cross validation (RMSECV) to determine the optimum number of principal factors. 

#### 2.6.2. Successive Projections Algorithm-Multiple Linear Regression (SPA-MLR)

Araujo et al. [37] first proposed the selection of spectral variables by means of the successive projections algorithm (SPA). Soares [38] used SPA for cross-classification analysis. The SPA, a forward variable selection method, uses vector projection analysis to find the variable group with minimal redundancy information to effectively eliminate the collinear, singular and instable variables in the spectra. Since it reduces the number of variables used in the model and lowers the complexity of the model, the collinear between the vectors is minimized. The multiple liner regression (MLR) adopts the least squares method to estimate the coefficient matrix, resulting in the samples whose numbers are more than the number of spectral variables. Extracting feature wavelength modeling based on SPA-MLR has significance in actual detection because of the useful information for mining spectral data with latent variables [39].

#### 2.6.3. Competitive Adaptive Weighting Method (CARS)

The competitive adaptive weighted algorithm method, imitating the evolution of “survival of the fittest” principle, phases out of the invariable wavelength [40]. It uses Monte Carlo sampling or random sampling method to select a part of the sample from the calibration set samples for PLS modeling and repeats this process for hundreds of iterations. In the process of wavelength variable selection, the adaptive weighted sampling method is used to preserve the wavelength variable with the absolute value of PLS regression coefficient, and the wavelength invariable with small absolute value of regression coefficient is removed. In order to obtain a series of wavelength variable subsets, each subset of wavelength variables is modeled by cross validation, and the optimal wavelength variable subset is selected according to the RMSECV value [41].

### 2.7. Model Evaluation Index

In this experiment, the modeling effect is evaluated by the correlation coefficient R, the root mean square error (RMSE) and the residual predictive deviation (RPD). The correlation coefficient R reflects the level of intimacy between variables, root mean square error (RMSE) reflects the accuracy of the model, and RPD reflects the prediction ability of the model. The higher the R and RPD and the lower the RMSE, the better the performance of the prediction model. In this paper, R_c_ and R_p_ represent the correlation coefficient of calibration set and prediction set, respectively, and RMSEC and RMSEP represent the root mean square error of the calibration set and prediction set respectively. Besides this, RPD was suggested to be at least 3 for agriculture applications; 2 < RPD < 3 indicates a model with a good prediction ability; 1.4 < RPD < 2 is an intermediate model needing some improvement; and the RPD < 1.4 indicates a poor prediction ability of the model [42].

## 3. Results and Discussion

### 3.1. Temperature and Soil Reflectance

**Figure 2:**
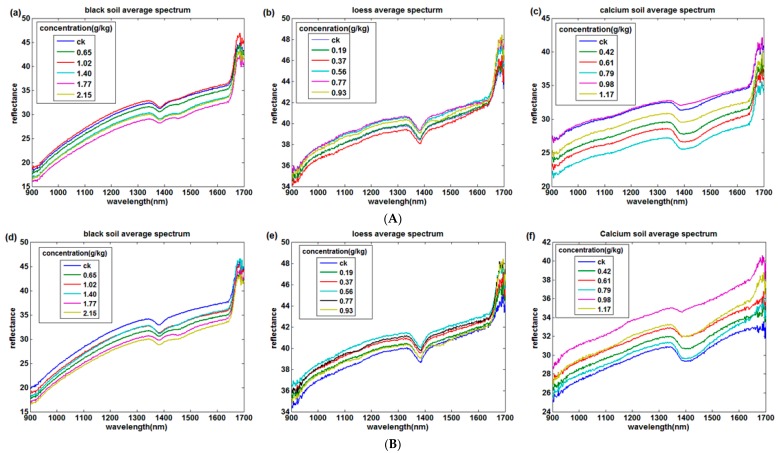

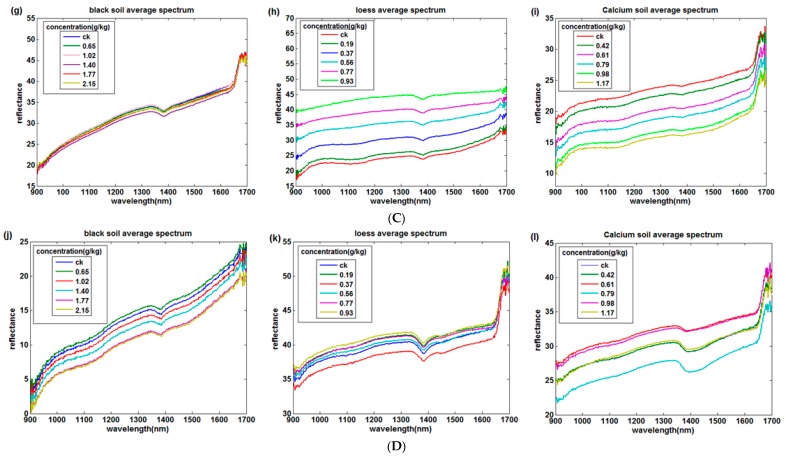
Near infrared spectra of three kinds of soils (**A**) 50 °C drying; (**B**) 80 °C drying; (**C**) 95 °C drying; (**D**) 25 °C placement. (**a**,**d**,**g**,**j**) are the black soil average spectrum at 50 °C, 80 °C, 95 °C drying and 25 °C placement respectively; (**b**,**e**,**h**,**k**) are the loess average spectrum at 50 °C, 80 °C, 95 °C drying and 25 °C placement respectively; (**c**,**f**,**j**,**l**) are the calcium soil average spectrum at 50 °C, 80 °C, 95 °C drying and 25 °C placement respectively.

In this experiment, the spectral information of three kinds of soil samples at four temperatures were collected. According to Figure 2, the abscissa of the curve is the wavelength and the ordinate of the curve is the average spectral reflectance.

Figure 2A–D shows the near infrared reflectance curves of the four soils after 50 °C, 80 °C, 95 °C drying and 25 °C placement respectively. First, the near infrared spectra of different soils vary from each other, but the overall trends are similar. The physical properties, chemical properties and soil colors would have certain influence on the absorption of near infrared spectra [43], which results in the differences of spectral curves. 

Second, the temperature does affect reflectance strength. The reflectance of the black soil spectral curve at 25 °C placement is significantly lower than other temperatures. The reason is that the water in the soil cannot be completely dried when soil was placed at 25 °C and the water absorption of near-infrared spectroscopy is very sensitive. The loess spectral curves are less affected by temperature because the loess are relatively loose, and porous, thus the water content in loess are easy to evaporate while drying. 

Third, the spectral absorption characteristics of those three soils are different. There is an obvious decrease trend in the band 1385 nm among the black soil, loess and calcium soil, which is caused by the vibration of O–H [44]. However, different soils have different characteristic bands. It is suggested in Figure 2a that the spectral reflectance of black soil decreases gradually at 1470 nm with the increase of nitrogen concentration of soil. Figure 2h shows that the spectral reflectance of loess decreases weakly near the band 1160 nm and Figure 2i displays that calcium soil has a spectral absorption at band 1145 nm when the drying temperature is 95 °C. Those mentioned above might be the characteristic bands of soil total nitrogen in different kinds of soils.

### 3.2. Data Modeling Prediction and Analysis

#### 3.2.1. SPA-MLR Model

**Figure 3:**
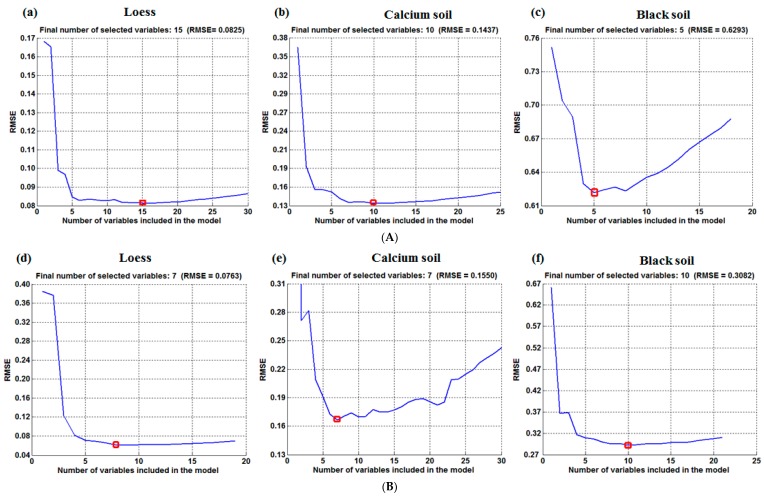

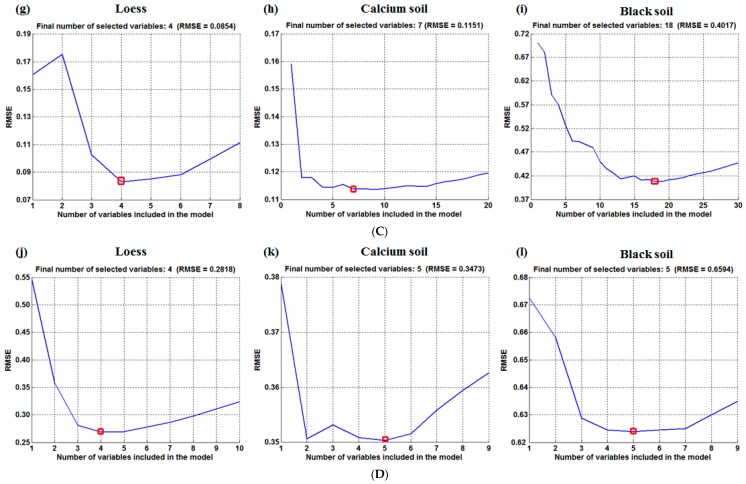
The wavelength number of loess, calcium and black soil selected by SPA: (**A**) 50 °C drying; (**B**) 80 °C drying; (**C**) 95 °C drying; (**D**) 25 °C placement. (**a**,**d**,**g,j**) are the loess wavelength number at 50 °C, 80 °C, 95 °C drying and 25 °C placement respectively; (**b**,**e**,**h**,**k**) are the calcium wavelength number at 50 °C, 80 °C, 95 °C drying and 25 °C placement respectively; (**c**),(**f**),(**j**) and (**l**) are the black soil wavelength number at 50 °C, 80 °C, 95 °C drying and 25 °C placement respectively.

The maximum number of selected variables was set up to 30, and the wavelength variables were selected from the 400 spectral variables based on the minimum error, which are shown in Table 1. Figure 3 presents the SPA wavelength number of loess, calcium soil and black soil, where Figure 3A–D represents the variable number of SPA after soil 50 °C, 80 °C, 95 °C drying and 25 °C placement respectively. It is indicated that although the variable numbers and bands differed in the same soil at different temperatures after the variable selection, the characteristic bands are similar when the temperatures varied small. The variable numbers and bands for different soils on the same drying temperature are not the same, suggesting that both soil type and drying temperature have a great influence on wavelength variables selected by SPA-MLR.

**Figure 4:**
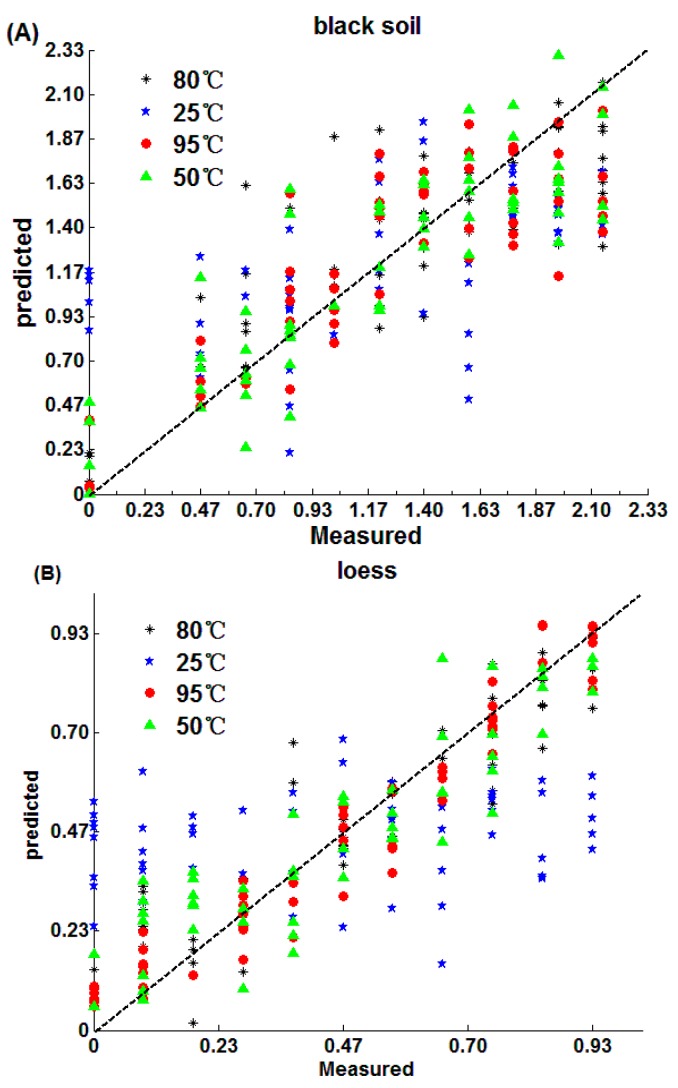

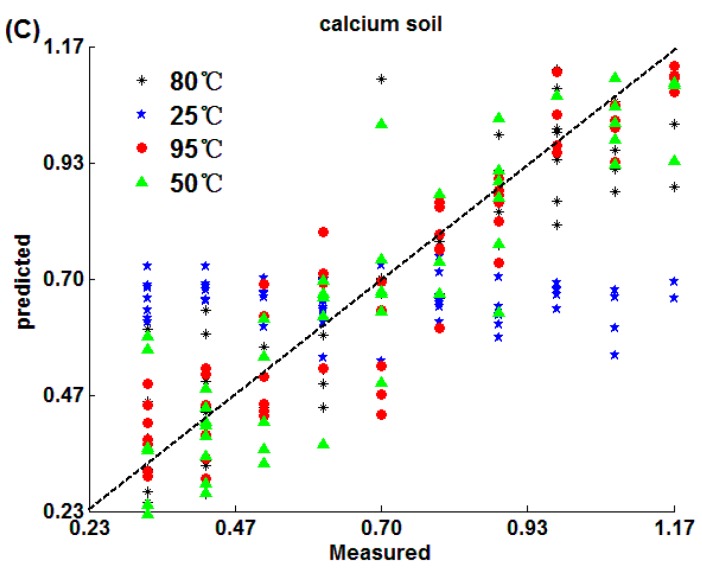
SPA-MLR algorithm prediction results: (**A**) black soil; (**B**) loess; (**C**) calcium soil.

The prediction results of SPA-MLR are shown in Table 2 and Figure 4. Both loess (R_P_ = 0.9758, RMSEP = 0.07 g/kg, RPD = 4.35) and calcium soil (R_P_ = 0.9517, RMSEP = 0.103 g/kg, RPD = 3.24) obtain the best detection effect after 95 °C drying, while black soil has a better detection effect after 50 °C (R_P_ = 0.9486, RMSEP = 0.22 g/kg, RPD = 2.82) and 80 °C(R_P_ = 0.9373, RMSEP = 0.234 g/kg, RPD = 2.55) drying, and the three kinds of soils have the worst effect when soils were placed in the 25 °C environment. 

On the one hand, the reason might be that medium and high temperature could stimulate the activity of soil urease and remove fully water in soil [45]. Meanwhile, soil water content was preserved little when soils were placed at 25 °C during the long time. Compared with O–H bond, the N–H bond exists mostly in multiple frequency or combination frequency, which was relatively weak in soil spectra and affects the extraction of soil nitrogen information [46]. 

On the other hand, the information of physical and chemical properties, including iron oxides, particle size distribution and surface roughness vary dramatically in different soils, which reduce or even obscure the spectral effect of nitrogen in soil [43]. Among them, loess mainly contains SiO_2_, Al_2_O_3_ and CaO with the properties of being loose and porous, and calcium soil mainly consisted of CaCO_3_. Both loess and calcium soil have few O–H bonds when they were dried, which interferes with the NIR spectrum to a small extent [47].

Hence, the prediction accuracy of loess and calcium soil was the optimum among three kinds of soils. However, the black soil obtains relatively low prediction accuracy because the abundant organic matter and humus in black soil have a strong absorption in NIR, resulting in adverse interference for nitrogen detection [48].

Figure 4 indicates that black soil nitrogen detection ranges from 0.93 g/kg to 1.87 g/kg, and the detection of nitrogen in loess is concentrated in the vicinity of 0.47 g/kg, while the soil nitrogen calcium concentrates from 0.47 g/kg to 0.93 g/kg when soils were placed at 25 °C, which largely deviates from the true values of the nitrogen in soil. The reason might be that the water content in soil was relatively higher when it was placed at 25 °C than when drying at other medium and high temperatures, which affects the extraction of soil nitrogen information [49].

#### 3.2.2. PLS Method Model 

**Figure 5:**
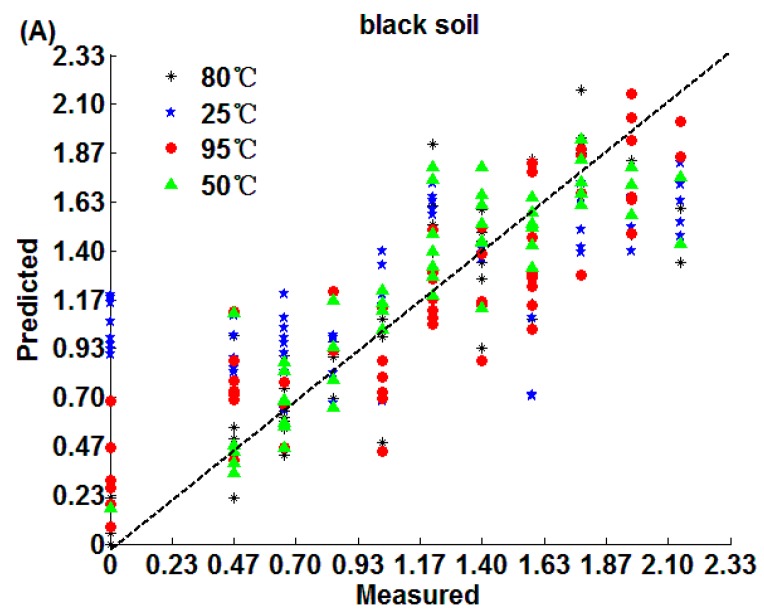

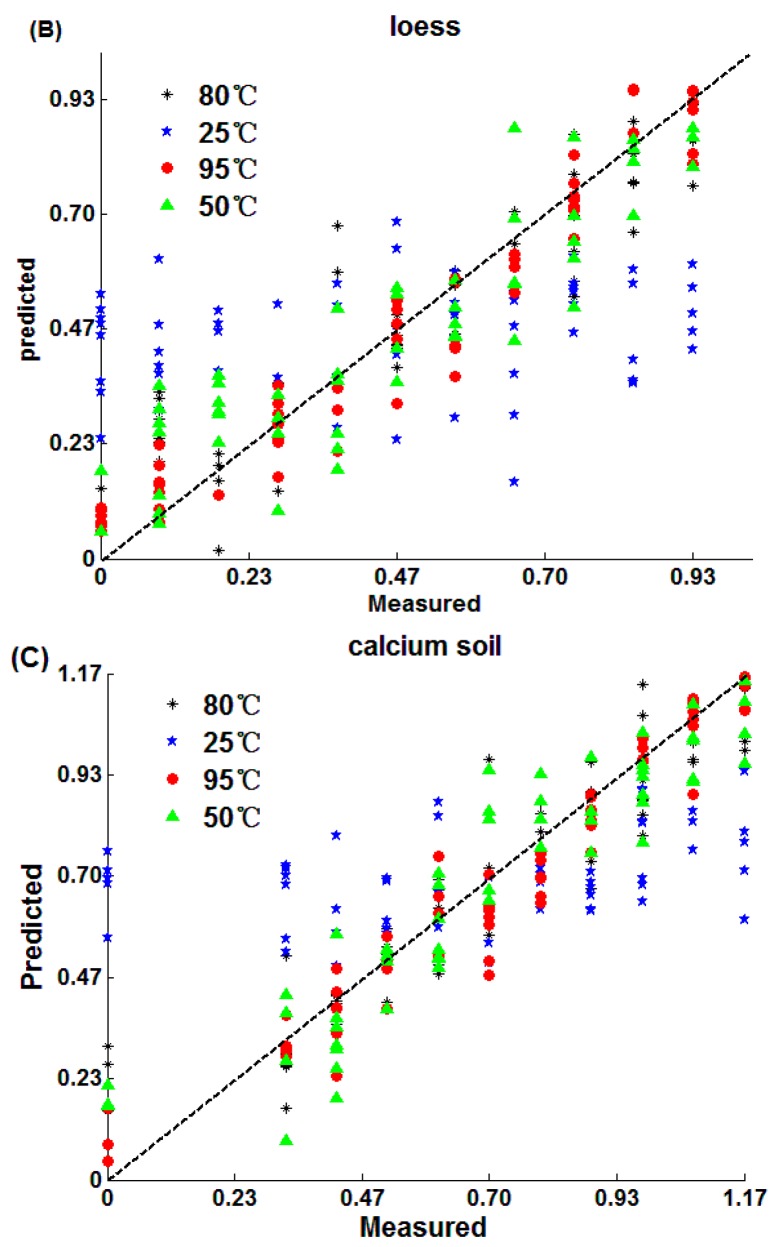
The prediction effect by PLS: (**A**) black soil; (**B**) loess; (**C**) calcium soil.

The prediction results of PLS are shown in Table 3 and Figure 5. The detection accuracy from high to low is loess, calcium soil and black soil, in that order. Moreover, both loess (R_P_ = 0.9721, RMSEP = 0.067 g/kg, RPD = 4.34) and calcium soil (R_P_ = 0.9588, RMSEP = 0.094 g/kg, RPD = 3.89) have the best detection accuracy after 95 °C drying. However, the black soil (R_P_ = 0.9216, RMSEP = 0.228 g/kg, RPD = 2.72) after 50 °C drying achieves the best detection accuracy of black soil. Moreover, the results of PLS and SPA-MLR are similar, which indicates that medium and high temperatures are helpful for the soil nitrogen detection and the reasons have been discussed in Section 3.2.1.

#### 3.2.3. CARS Model Methods

**Figure 6:**
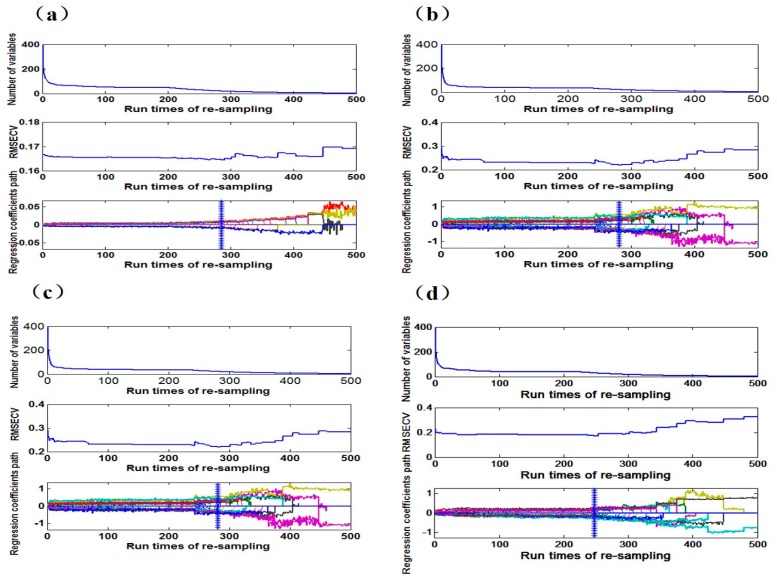
The variable selection process by competitive adaptive reweighted squares (CARS): (**a**) 50 °C drying; (**b**) 80 °C drying; (**c**) 95 °C drying; (**d**) 25 °C placement.

The setting times of the CARS variable selection was 500, and the variable selection process are shown in Figure 6. Figure 6a–d show the variable selection process of loess after 50 °C, 80 °C and 95 °C drying and 25 °C placement, respectively. The number of final variable numbers and principal components are shown in Table 4. After the selection, the number of variables and bands differ in the same soil at different drying temperatures and the number of variables and bands in different soil types on the same drying temperature vary from each other as well, indicating that both soil type and soil drying temperature have a great influence on wavelength variables selected by CARS.

**Figure 7:**
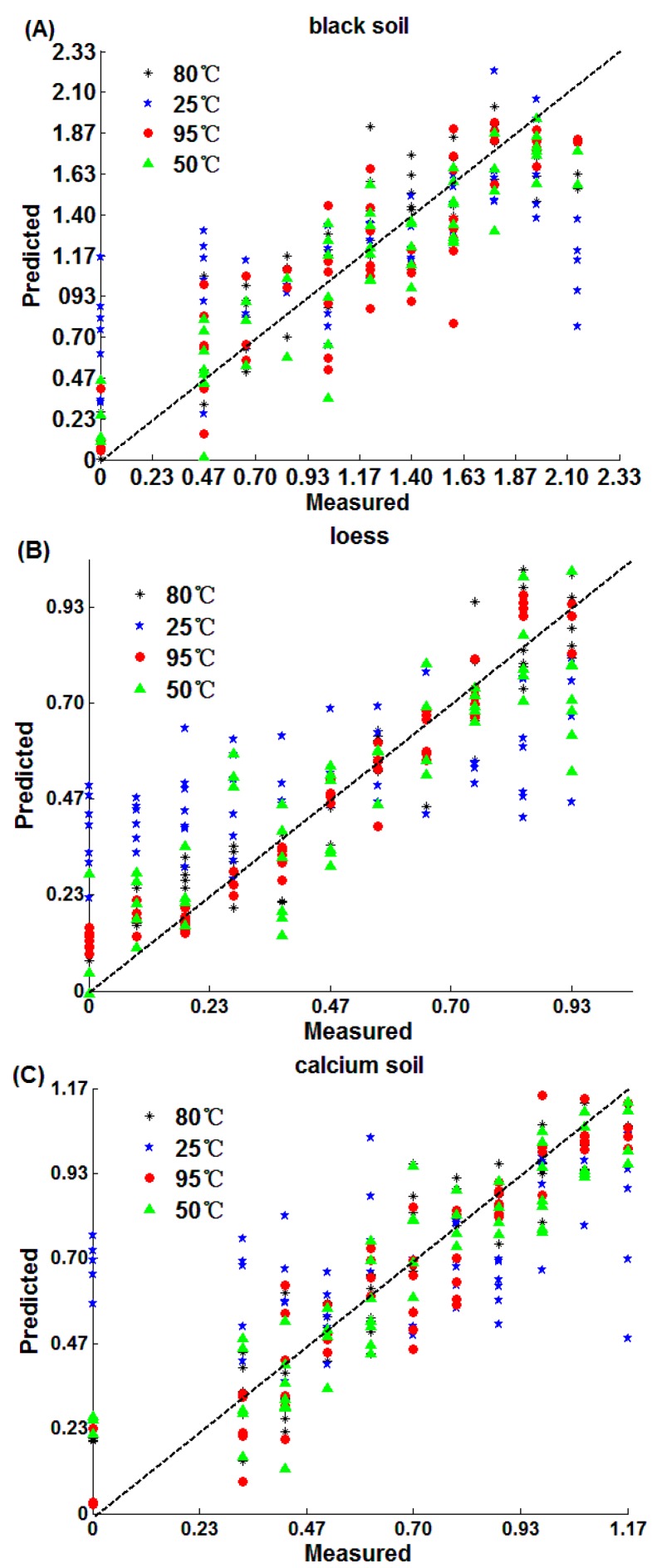
CARS prediction results: (**A**) black soil; (**B**) loess; (**C**) calcium soil.

The prediction results of the CARS are shown in Figure 7 and Table 5. The results of nitrogen prediction of loess (R_P_ = 0.9612, RMSEP = 0.079 g/kg, RPD = 3.92) and calcium soil (R_P_ = 0.9472, RMSEP = 0.112 g/kg, RPD = 3.07) are the best after 95 °C drying. While the black nitrogen prediction was best after 80 °C drying. Also, the three kinds of soils have the worst effects when soils were placed at 25 °C. The results are similar to the SPA-MLR and PLS, which indicates that medium and high temperatures are beneficial to soil nitrogen detection and the reasons have been discussed in Section 3.2.1. 

### 3.3. Analysis and Comparison of Results

**Figure 8:**
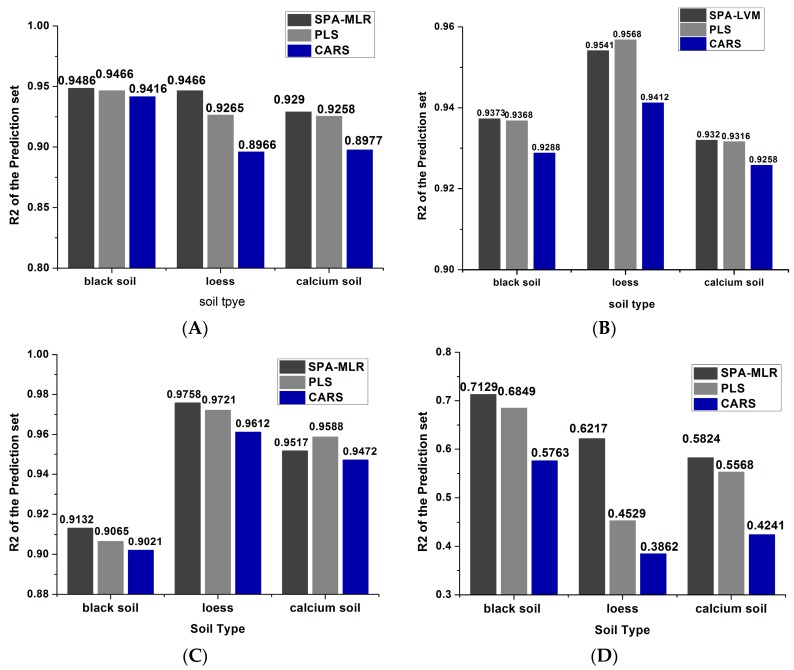
The prediction results of three kinds of soils at different drying temperatures based on three algorithms: (**A**) 50 °C drying; (**B**) 80 °C drying; (**C**) 95 °C drying; (**D**) 25 °C placement.

Figure 8 shows the results of different models of three kinds of soils at different temperatures. First, the soil nitrogen detection effect ranking of loess and calcium soil was 95 °C, 80 °C, 50 °C and 25 °C, while in black soil, the effect of soil nitrogen detection was 50 °C, 80 °C, 95 °C and 25 °C, in that order. 

This indicated that urease in soil was activated and the water content was easier to fully evaporate under medium and high drying temperatures, thus the detection effects were obviously better. However, the information of physical and chemical properties including iron oxides and particle size distribution were different in soil [43], which caused the different detection results when different soils were dried at the same temperature. Moreover, the prediction effects of black soil in 50 °C, 85 °C and 90 °C were worse than that of the loess and the calcium soil, but the results were better than those of other soils when soils were placed at 25 °C, which not only indicated that the physicochemical properties of the black soil caused the difference of the results, but also suggested that the little water preserved in the drying process had the least influence on the nitrogen detection in black soil than other soils.

Second, different algorithms had different effects on soil nitrogen detection based on the same temperature. The overall detection effect ranking from better to worse was SPA-MLR, PLS and CARS, and the prediction accuracy of SPA-MLR and PLS was similar. The reason might be that SPA-MLR could efficiently eliminate redundant variables, which made the results more accurate and the detection precision higher [39]. The comprehensive variables extracted by PLS performed well in summarizing the information of independent variables, explaining dependent variables and eliminating noise interference in the system, which effectively handled the variables multiple correlation problem [42]. In the CARS, it was difficult to find the best or optimal value of the noise threshold and select the randomness of the characteristic variables, which leaded to the poor prediction results [41].

**Figure 9:**
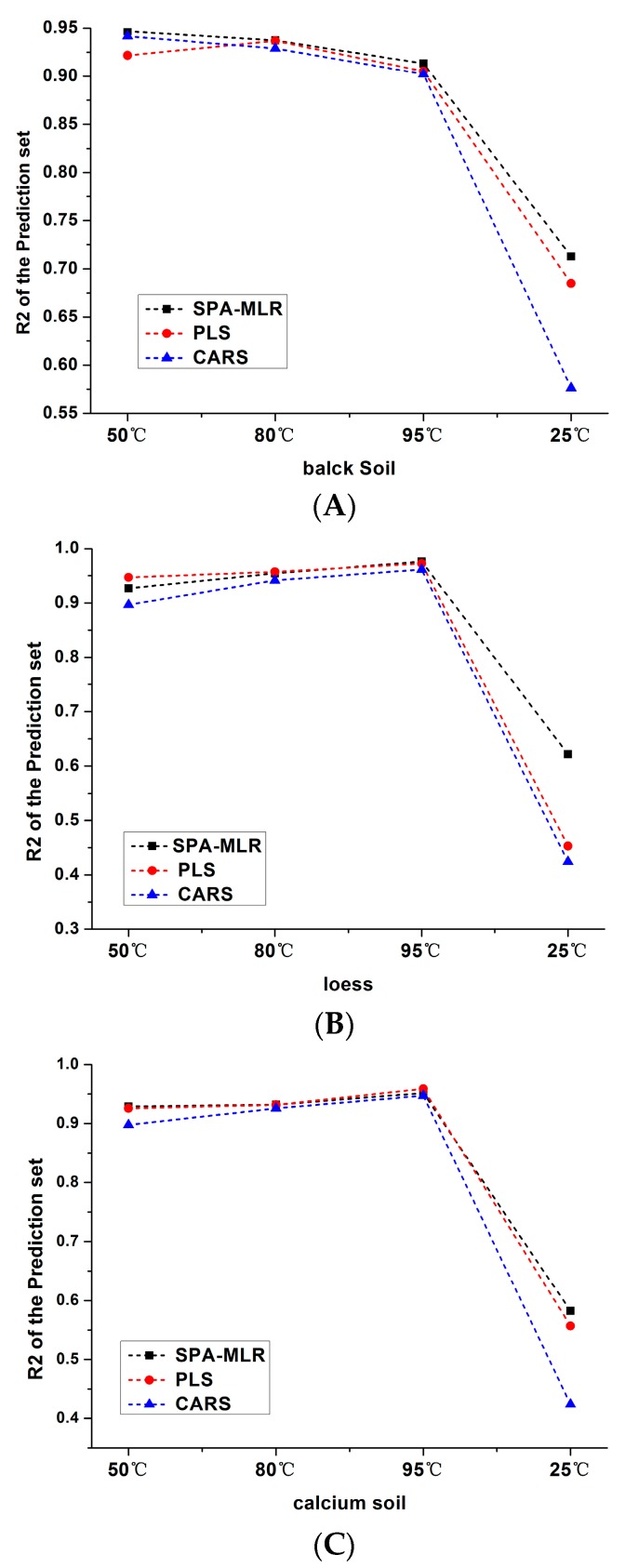
Prediction results of three kinds of soil based on different temperatures algorithms: (**A**) black soil; (**B**) loess; (**C**) calcium soil.

Figure 9 shows the prediction effects of soils under different drying temperatures using different algorithms. 

As can be seen from the Figure 9, no matter which algorithm was used, the prediction accuracy of black soil was better when the drying temperatures ranged from 50 °C to 80 °C. While the detection accuracy of loess and calcium soil nitrogen obtained better results when the temperatures were in the range of 80 °C to 90 °C.

## 4. Conclusions

In this paper, three kinds of soils were used to investigate the effects of drying temperature on soil nitrogen detection using near infrared sensors. The NIR spectra of different soils varied greatly and the spectra of the same soil type changed greatly under different drying temperatures. 

The main conclusions are as follows: (1) The drying temperature does have an influence on soil nitrogen detection by near infrared sensors and the suitable drying temperatures for different soils were different, which indicated that it was necessary to find the suitable drying temperature for different soil types to enhance the detection accuracy; (2) the drying temperatures ranged from 50 °C to 80 °C for black soil nitrogen detection accuracy were better than other temperatures, while the loess and calcium soil nitrogen detection had better results when the drying temperature was 95 °C. The O–H bonds of water in soil might be the main factor influencing the prediction accuracy when soils were placed at 25 °C. Besides this, the suitable drying temperatures for more soil types should be further researched; (3) the SPA-MLR and PLS models obtained a better prediction effect for soils, while CARS performed worse. In conclusion, drying temperature had an obvious influence on the detection of soil nitrogen by near infrared sensors, and the suitable drying temperature for different soil types was of great significance in enhancing the detection accuracy.

## Figures and Tables

**Table 1:** Successive projections algorithm based on multiple linear regression (SPA-MLR) algorithm variable number and wavelength.

Soil Type	Temperature	Variable Number	Wavelength (nm)
Loess	50 °C	15	915, 1428, 1695, 1694, 1693, 1692, 1487, 1550, 1683, 1676, 1673, 1675, 1686, 1582, 1650
	80 °C	7	1160, 1660, 1582, 1682, 1675, 1489,1428
	95 °C	4	1160, 1428, 1675, 1486
	25 °C	4	1424, 1488, 1694, 1428
Calcium soil	50 °C	10	1651, 1154, 1438, 910,1301, 979, 1450, 1675, 1246, 1677
	80 °C	7	1651, 1675, 1678, 979, 1677, 1058, 1244
	95 °C	7	1552, 1675, 1487, 1491, 1673, 921, 1650
	25 °C	5	1651, 1675, 1146, 979, 1167
Black soil	50 °C	5	1423, 928, 1654, 1496, 1694
	80 °C	10	928, 1654, 1681, 1682, 1694, 1496, 1423, 915, 1684, 1662
	95 °C	18	1650, 1680, 1682, 1694, 915, 1684, 1050, 1429, 1491, 1662, 928, 925, 910, 916, 918, 1662, 1675, 1690
	25 °C	5	1423, 925, 1681, 1496, 1694

**Table 2:** The modeling results of different soils and temperatures by SPA-MLR. RMSEC: root mean square error (RMSE) of the calibration set; RMSEP: RMSE of the prediction set; RPD: residual predictive deviation.

Group	Soil Type	Calibration Set			Prediction Set			
		N1	R_c_	RMSEC (g/kg)	N2	R_p_	RMSEP (g/kg)	RPD
1 (50 °C)	Black soil	118	0.9725	0.11	58	0.9486	0.22	2.82
	Loess	118	0.9649	0.072	58	0.9265	0.13	2.34
	Calcium soil	118	0.9681	0.039	58	0.9290	0.120	2.63
2 (80 °C)	Black soil	118	0.9203	0.251	58	0.9373	0.234	2.55
	Loess	118	0.9727	0.067	58	0.9541	0.090	3.20
	Calcium soil	118	0.9492	0.108	58	0.9320	0.162	2.12
3 (95 °C)	Black soil	118	0.9692	0.156	58	0.9132	0.282	2.16
	Loess	118	0.9660	0.075	58	0.9758	0.070	4.35
	Calcium soil	118	0.9670	0.087	58	0.9517	0.103	3.24
4 (25 °C)	Black soil	118	0.6061	0.486	58	0.7129	0.418	1.25
	Loess	118	0.5473	0.247	58	0.6217	0.246	1.20
	Calcium soil	118	0.4391	0.302	58	0.5824	0.365	0.94

**Table 3:** The modeling results of different soil types and temperatures by partial least squares (PLS).

Group	Soil Type		Calibration Set			Prediction Set		
		N1	R_c_	RMSEC (g/kg)	N2	R_p_	RMSEP (g/kg)	RPD
1 (50 °C)	Black soil	118	0.9525	0.198	58	0.9216	0.228	2.72
	Loess	118	0.9609	0.077	58	0.9466	0.112	2.71
	Calcium soil	118	0.9881	0.057	58	0.9258	0.128	2.69
2 (80 °C)	Black soil	118	0.9417	0.216	58	0.9368	0.217	2.82
	Loess	118	0.9935	0.033	58	0.9568	0.090	3.31
	Calcium soil	118	0.9173	0.132	58	0.9316	0.119	2.75
3 (95 °C)	Black soil	118	0.9906	0.086	58	0.9065	0.273	2.22
	Loess	118	0.9739	0.066	58	0.9721	0.067	4.34
	Calcium soil	118	0.9269	0.129	58	0.9588	0.094	3.89
4 (25 °C)	Black soil	118	0.7773	0.391	58	0.6849	0.480	1.26
	Loess	118	0.3507	0.267	58	0.4529	0.287	1.09
	Calcium soil	118	0.5332	0.286	58	0.5568	0.258	1.34

**Table 4:** The selected variables and principal component number.

Soil Type	Temperature	Selected Variables Number	Principal Component Number
Loess	50 °C	20	5
	80 °C	40	6
	95 °C	19	3
	25 °C	29	3
Calcium soil	50 °C	18	3
	80 °C	26	5
	95 °C	20	5
	25 °C	14	3
Black soil	50 °C	21	5
	80 °C	11	5
	95 °C	42	6
	25 °C	32	5

**Table 5:** The modeling results of different soils and temperatures by CARS.

Group	Soil Type		Calibration Set			Prediction Set		
		N1	R_c_	RMSEC (g/kg)	N2	R_p_	RMSEP (g/kg)	RPD
1 (50 °C)	Black soil	118	0.9625	0.163	58	0.9416	0.185	2.95
	Loess	118	0.9009	0.1875	58	0.8966	0.1885	1.61
	Calcium soil	118	0.9281	0.1549	58	0.8977	0.1414	2.43
2 (80 °C)	Black soil	118	0.9205	0.25	58	0.9288	0.237	2.68
	Loess	118	0.93	0.106	58	0.9412	0.105	2.90
	Calcium soil	118	0.9117	0.136	58	0.9258	0.119	2.79
3 (95 °C)	Black soil	118	0.9731	0.146	58	0.9021	0.277	2.24
	Loess	118	0.9609	0.077	58	0.9612	0.079	3.92
	Calcium soil	118	0.9381	0.118	58	0.9472	0.112	3.07
4 (25 °C)	Black soil	118	0.5458	0.551	58	0.5763	0.476	1.31
	Loess	118	0.5615	0.247	58	0.3862	0.265	1.15
	Calcium soil	118	0.3698	0.322	58	0.4241	0.304	1.13
